# Insight Into Nicotinamide Adenine Dinucleotide Homeostasis as a Targetable Metabolic Pathway in Colorectal Cancer

**DOI:** 10.3389/fphar.2021.758320

**Published:** 2021-11-22

**Authors:** Giorgia Colombo, Edoardo Luigi Maria Gelardi, Federica Carolina Balestrero, Marianna Moro, Cristina Travelli, Armando A. Genazzani

**Affiliations:** ^1^ Department of Pharmaceutical Sciences, Università Del Piemonte Orientale, Novara, Italy; ^2^ Department of Experimental Oncology, European Institute of Oncology IRCCS, Milan, Italy; ^3^ Department of Drug Sciences, Università Degli Studi di Pavia, Pavia, Italy

**Keywords:** NAD, NADPH, NAMPT (nicotinamide phosphoribosyltransferase), isocitrate dehydrogenase (IDH), CD38, ALDH = aldehyde dehydrogenase, PARP, sirtuins

## Abstract

Tumour cells modify their cellular metabolism with the aim to sustain uncontrolled proliferation. *Cancer* cells necessitate adequate amounts of NAD and NADPH to support several enzymes that are usually overexpressed and/or overactivated. Nicotinamide adenine dinucleotide (NAD) is an essential cofactor and substrate of several NAD-consuming enzymes, such as PARPs and sirtuins, while NADPH is important in the regulation of the redox status in cells. The present review explores the rationale for targeting the key enzymes that maintain the cellular NAD/NADPH pool in colorectal cancer and the enzymes that consume or use NADP(H).

## Introduction

Colorectal cancer (CRC), a frequent cancer that occurs in both males and females (Kim HI. et al., 2018), is characterized by an high rate of mortality (Brody, 2015). Overall, 5-years survival rates are high, around 90%, but this percentage decreases to 66% in patients with regional lesions and metastasis (Koyanagi et al., 2008). From a therapeutic point of view, new drugs have extended the survival rate, but there is still a strong unmet medical need in advanced or metastatic CRC, in particular due to chemotherapy/target therapy resistance and unresponsiveness to new therapies (Brody, 2015; Loree and Kopetz, 2017). Finding new therapeutic strategies in CRC is therefore both challenging and necessary.

Among the cancer features that define aggressiveness, there is the reprogramming of cellular metabolism (Loree and Kopetz, 2017). Abnormal consumption of glucose and increased production of lactate, with a subsequent decrease of oxidative phosphorylation through aerobic glycolysis, is defined as the “Warburg effect”, and allows cancer cells to win the evolutionary game of the “survival of the fittest” thereby allowing them to proliferate both in aerobic and anaerobic environments (Vander Heiden et al., 2009). Therefore, also gluconeogenesis, that is opposed to aerobic glycolysis, is able to hijack cell plasticity, promoting tumour growth (Grasmann et al., 2019). Nicotinamide adenine dinucleotide (NAD) and nicotinamide adenine dinucleotide phosphate (NADPH) are indispensable in regulating these metabolic reactions as well as for adenosine triphosphate (ATP) production. While the ubiquitous nature of these pathways renders their pharmacological targeting challenging, a number of groups have now focused their attention on this possibility. These strategies can be sub-divided in distinct chapters: 1) reducing the NAD(H) supply of cells by inhibiting the enzymes involved in its synthesis, thereby reducing the activity of all down-stream pathways dependent on this molecule; 2) selectively inhibiting NAD-consuming enzymes: 3) selectively inhibiting enzymes that use NAD(P)(H) as a co-factor.

Several reviews have addressed the advantage of targeting NAD/NADPH homeostasis and consuming enzymes in cancer (Audrito et al., 2019; Galli et al., 2020; Ju et al., 2020; Rather et al., 2021); thus, an extensive description is beyond the purpose of this manuscript, and we refer to other excellent reviews on the subject, including the ones that describe the entire NADome (Chiarugi et al., 2012). In the present review, we have searched on MEDLINE/PubMed the names more than of 400 NAD and NADPH-dependent enzymes followed by the term “colorectal cancer” and have chosen the most relevant, and less reviewed, targets in the context of CRC. In particular, we have included papers pointing out targets in a pharmacological perspective, avoiding results of proteins found exclusively in sequencing analysis.

**FIGURE 1:**
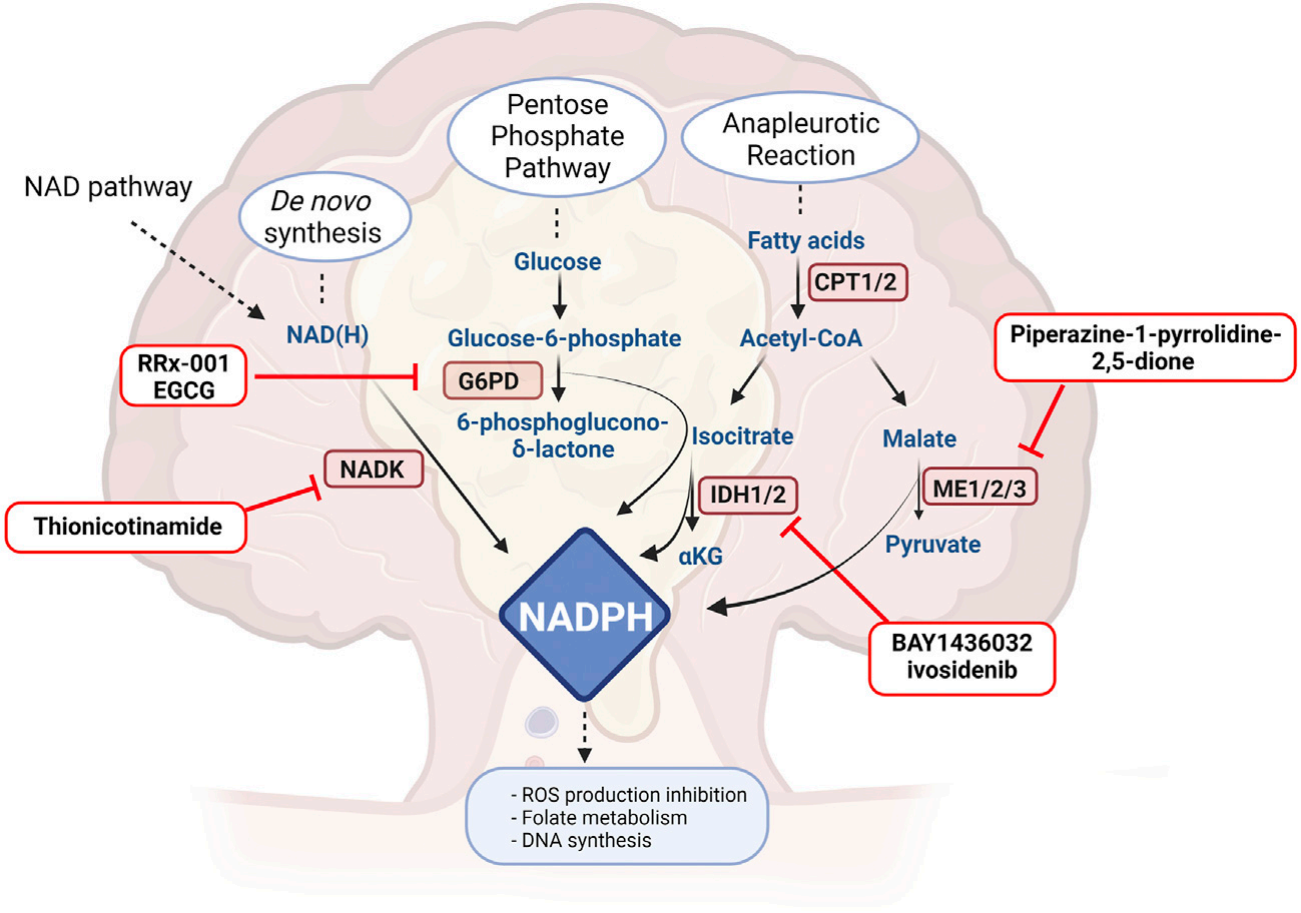
Drugs or molecules that have been postulated as effective in CRC and that target NAD routes. NAMPT: nicotinamide phosphoribosyltransferase, NAPRT: nicotinic acid phosphoribosyltransferase, NMNAT: nicotinamide mononucleotide adenylyltrasnferase, NADSYN: NAD synthetase, PARP: Poly-ADP ribose polymerase, ALDHs: aldehyde dehydrogenase. Created with BioRender.com.

NAD homeostasis results from the balance between NAD-consuming reactions and NAD-biosynthetic routes. Indeed, NAD-consuming enzymes (*e.g*., poly-ADP-ribose polymerases (PARPs)) are upregulated and have a higher activity in cancer, and metabolic enzymes that require NAD(P) as a co-factor are also up-regulated, resulting in a higher demand of these molecules which is provided by NAD-synthetic paths. It is therefore not surprising that several manuscripts have investigated the effect of inhibition of these pathways in CRC, as summarized in Figure 1.

## Nicotinamide Adenine Dinucleotide Synthesis as a Target

There are four main NAD biosynthetic pathways in eukariotic cells: *1*) the *de novo* biosynthetic *Pathway* from quinolinic acid, derived from dietary tryptophan, *2*) the *Preiss-Handler Pathway* (PH-pathway) from nicotinic acid (NA), *3*) the *Salvage Pathway* from nicotinamide (NAM), mediated by nicotinamide phosphoribosyltransferase (NAMPT) and *4*) the newly described route from nicotinamide riboside (Tempel et al., 2007) through nicotinamide riboside kinase (NMRK). These four pathways converge in the production of a mononucleotide, catalysed by different phosphoribosyltransferase enzymes depending on the starting precursor. In the last step, which is common to all routes, the formation of NAD is catalysed by nicotinamide mononucleotide adenylyltransferase (NMNAT). Nicotinamide is considered the main precursor of NAD due to its availability compared to nicotinic acid: it is present in higher concentrations in the bloodstream, it is easily introduced by diet, and it is the reaction product of NAD-dependent enzymes.

Due to the Warburg effect and to over-activation of NAD-consuming enzymes, proliferating cells necessitate larger NAD supplies (La Vecchia and Sebastián, 2020). Chowdhry et al. have highlighted that tumours are usually dependent on the PH-pathway (PH-dependent tumours) or from salvage pathway (salvage-dependent tumours). In general, this is usually linked to whether the original healthy tissue expressed higher levels of either nicotinic acid phosphoribosyltransferase (NAPRT) or NAMPT (Chowdhry et al., 2019). While these two enzymes have received considerable attention in cancer, every enzyme that sustains NAD production might be a possible target for targeting CRC.

### NAMPT and NAPRT as Drug Targets in Colorectal Cancer

Nicotinamide phosphoribosyltransferase is the key enzyme of the NAD Salvage Pathway and belongs to type II phosphoribosyltransferases. It exists as a dimer in two different forms: an intracellular form (iNAMPT) that is localized in the cytosol and in the nucleus, and an extracellular form (eNAMPT) that is secreted in extracellular fluids by various cell types. NAPRT is structurally similar and has also been reported to be both intracellular and extracellular.

iNAMPT is situated inside the cell as a homodimer with a molecular weight of about 110 kDa. It is involved in NAD biosynthesis and catalyses the production of nicotinamide mononucleotide (NMN) from NAM and 5-phosphoribosyl-1-pyrophosphate (PRPP). Since it controls the intracellular levels of NAD (*i.e.,* its inhibition leads to a significant drop in levels), iNAMPT modulates the activity of NAD-dependent enzymes, such as PARPs and sirtuins (SIRTs). NAPRT, instead, uses NA as a substrate.

These two enzymes therefore run parallel routes of synthesis. According to Chowdhry et al., colon cancer cells appear to be NAMPT-dependent, due to lower levels of NAPRT in the primary tissue (Chowdhry et al., 2019), even though other manuscripts reveal a dependence also from NAPRT (Hong et al., 2019) and a higher expression of this latter enzyme in healthy small intestine, colon cancer cell lines and biopsies (Hara et al., 2007; Duarte-Pereira et al., 2016; Cole et al., 2017).

While a number of drug discovery programs have initiated to develop NAPRT inhibitors, potent and selective ligands have so far been elusive, and it is therefore not known whether NAPRT inhibition may be a viable strategy to target CRC. On the contrary, the field of iNAMPT has capitalized on the report, back in 2003, of the first specific nanomolar inhibitor, FK866 or APO866, which has been followed by a number of other inhibitors which have either reached clinical stages (CHS828, GMX1777, KPT9274 and OT82) or have only been carried forward only at the preclinical level (Holen et al., 2008; Pishvaian et al., 2008; Goldinger et al., 2016; Galli et al., 2020).

Alongside the metabolic rationale, it is also known that iNAMPT overexpression is correlated to colorectal carcinoma rather than adenoma and it is therefore not surprising that its inhibition, using FK866, leads to cell death of a number of CRC cell lines, including HT29, HCT116 and Caco-2 (Lv et al., 2015). Similarly, the small molecule GMX1778, another inhibitor that has reached clinical trials, is able to induce antitumour responses in HCT-116 xenograft model (Watson et al., 2009). Similarly, another NAM-competitive inhibitor, named A-1293201, is also effective in the same model (Wilsbacher et al., 2017).

Tolstikov et al. have characterized iNAMPT inhibition in CRC, again using FK866 and mass-spectrometry-based metabolomics (Tolstikov et al., 2014). Using the human HCT116 cell line (Duke’s staging type D) and have reported how FK866 is able to hinder several metabolic pathways and their related signalling pathways. In brief, FK866 is able to 1) attenuate glycolysis and the pentose phosphate pathway and thereby lead the accumulation of glycolytic substrates; 2) attenuate nucleotide and amino acid metabolism with a subsequent reduction of purine and pyrimidine metabolism; 3) induce reduction of pyruvate entry in the TCA cycle with a consequent reduction in aspartate and alanine synthesis; 4) induce an attenuation of purine biosynthesis, with an accumulation of xanthine; and 5) reduce fatty acid and lipid metabolism (Tolstikov et al., 2014). Alongside metabolism, iNAMPT has also been shown to sustain neoplastic progression acting on cancer stem cells and Wnt/β-catenin signalling (Lucena-Cacace et al., 2018; Ye et al., 2020). Indeed, iNAMPT is now considered an oncogene, overexpressed in colon cancer and correlated to poor prognosis, able to promote tumour initiation through cancer initiating cells (CICs), in CICs xenograft using HCT116 and LS180 cell lines (Lucena-Cacace et al., 2018). This overexpression determines chemoresistance mediated by an increase in CD133 + cancer stem cells, overexpressing PARPs and SIRT1, and the treatment with FK866 in combination with olaparib (PARP inhibitor) or sirtinol (SIRT inhibitor) is able to counteract tumour progression (Lucena-Cacace et al., 2018). Moreover, iNAMPT regulates Wnt/β-catenin pathway promoting CRC growth, so using FK866, β-catenin nuclear translocation and Wnt/β-catenin target gene cyclin D1 are hampered. Axin, a component of the β-catenin demolition complex, is subsequently elevated after FK866 treatment (Ye et al., 2020). On the other hand, the resistance of FK866 may sensitise cells to 5-fluorouracil, cisplatin and γ-rays in HCT116-resistant cells (Ogino et al., 2018, 2019). Both iNAMPT and NAPRT have been found overexpressed in a model of cancer-associated colitis (CAC), as azoxymethane (AOM)/dextran sodium sulphate (DSS), highlighting a possible effect on inflammation that undergoes cancer development.

In 1994, a cytokine, referable to the extracellular form of NAMPT (eNAMPT), was identified as an immunomodulatory agent able to promote pre-B cell colony formation synergizing with IL-7 and stem cell factor (SCF) (Samal et al., 1994). Indeed, it has now been ascertained that eNAMPT can also be secreted by a number of different cell types (including cancer cells) and acts as a cytokine that modulates the immune response (Audrito et al., 2015; Camp et al., 2015). It is still unclear whether enzymatic activity is maintained and if it is necessary for its cytokine-like action (Grolla et al., 2016). iNAMPT and eNAMPT have the same amino-acid sequence and the same quaternary structure, but specific post-translational modifications might be responsible for eNAMPT secretion. In particular, it has been suggested that the deacetylation of lysine 53 on iNAMPT, operated by SIRT1, predisposes the protein to secretion and enhances eNAMPT activity in adipocytes (Yoon et al., 2015). No testimony certifies a possible effect of targeting eNAMPT in CRC, even though it has been found elevated in CRC patients (Nakajima et al., 2010), and shown to promote multi-drug resistance (Yan et al., 2017) and increase reactive oxygen species (ROS) (Buldak et al., 2015). Our group and Garcia’s have recently developed neutralizing monoclonal antibodies against eNAMPT (Colombo et al., 2020; Garcia et al., 2021), and it is possible that in the future data in CRC will be gathered also in this respect although the relationship between eNAMPT and NAD homeostasis is unclear. More recently, also NAPRT has been found in the extracellular space (eNAPRT), elevated in septic patients. Managò et al. have highlighted also its presence in some tumour patients, compared to healthy subjects, but more information needs to be gathered (Managò et al., 2019).

Overall, therefore, iNAMPT inhibition would appear as a plausible target. While efficacy would be supported by the above data, there are nonetheless doubts regarding safety, with a number of on-target side effects (Olesen et al., 2010; Tarrant et al., 2015), probably due to the ubiquitous nature of this enzyme. The verdict is not final, though, as there are dual agents (PAK4 p21-activated kinase and NAMPT dual inhibitor) in the clinic and a series of NAMPT inhibitors has been reported as not having these side effects in preclinical tests (Abu Aboud et al., 2016; Travelli et al., 2019a; Galli et al., 2020). Moreover, iNAMPT has appeared to be a promising target also in the immune counterpart of tumours. NAMPT inhibitors appear to have a role in hijacking myeloid-derived suppressor cell (MDSC) mobilization, activating antitumor immune responses and sensibilizing tumours to immune checkpoint inhibitors (Travelli et al., 2019b) as a result of a direct control of the microenvironment. No one has hypothesized this strategy in CRC, but it appears to be promising in CRCs with high immune infiltration or dependent on bowel inflammation (IBD-CRCs). This suggests that targeting iNAMPT in CRC could have a direct effect on tumours and an indirect anticancer activity on the immune system.

### Therapeutic role of NMNAT Targeting in Colorectal Cancer

The final products of iNAMPT and NAPRT, NMN and nicotinic acid mononucleotide, are then transformed in NAD and nicotinic acid adenine dinucleotide (NAAD) through NMNAT, which is present in cells in three different isoforms (NMNAT1,2,3). NMNAT1 is predominantly present in the nucleus, NMNAT2 in the Golgi apparatus (Berger et al., 2005), while NMNAT3 in mitochondria or cytoplasm (Cambronne and Kraus, 2020). NMNAT2 has been highlighted as a possible diagnostic target for CRC, as its levels correlate with p53 in more invasive tumours, even though it is not correlated with overall survival (Cui et al., 2016). Furthermore, NMNAT2 overexpression in CRC is linked to SIRT6 downregulation (Qi et al., 2018).

It has been found that overexpression of NMNAT2 sensitises Caco-2 and HT-29 to tiazofurin, inducing cell death, while low level-NMNAT2 cell lines appear to be refractory (Kusumanchi et al., 2013). This is most likely due to a link between the amount of NAD and the presence of inosine monophosphate dehydrogenase (IMPD), the target of the active metabolite of tiazofurin, which is an NAD analogue. While NMNAT2 upregulation favours tiazofurin action, which were abandoned in clinics due to adverse events (Popsavin et al., 2006), it is thought that inhibitors of this enzyme would be a significantly better therapeutic option. Yet, at present no potent NMNAT inhibitor has been developed (Petrelli et al., 2011; Buonvicino et al., 2018), with the exception of 2,3-dibromo-1,4-naphthoquinone (Haubrich et al., 2020), which nonetheless lacks selectivity against the different isoforms and possibly acts also on other targets. Yet, given the convergence of both iNAMPT and NAPRT on NMNAT2, it could be postulated that inhibitors of these isoform in CRC may be attractive.

## Nicotinamide Adenine Dinucleotide-Utilizing Enzymes as Drug Targets

### *Therapeutic Role of* Poly-ADP-Ribose Polymerase *Inhibitors in Colorectal Cancer*

Increased production of ROS in cancer cells determines DNA damage, with base lesions or single strand breaks (Saikolappan et al., 2019; Chang et al., 2020). While some of these might lead to pro-tumoural permissive mutations, an excess would invariably lead to apoptosis. This balance is maintained by PARPs, an 18-member nuclear enzyme family involved in DNA damage repair. PARPs orchestrate chromatin remodelling, transcription, replication, recombination and DNA repair (Morales et al., 2014) and are upregulated in CRC (Nosho et al., 2006; Dziaman et al., 2014). Of these 18 members, the most relevant ones in the context of cancer appear to be PARP1 and PARP2, recognized for their ability to activate base excision repair (BER) in response to single-stranded DNA breaks (SSBs). (Amé et al., 2004). Indeed, most tumours, to counterbalance ROS production, overexpress PARPs with the aim to increase genomic stability (Nomura et al., 2000). To support this, PARP-1 knock-out cells are more sensitive to exogenous DNA damage agents, such as alkylating drugs and irradiation (Menear et al., 2008). Briefly, in front of DNA damage response, there is the activation of several pathways that leads to poly ADP-ribosylation (PARylation) in the damage site by PARPs using NAD as a source of adenosine diphosphate ribose (ADPR).

Several PARP inhibitors have been developed and are already in the armamentarium of oncologists. Most of their indications at present pertain to patient populations (e.g., ovarian, breast, pancreatic, prostate cancer) in which there is a deficit of the BRCA1/2 genes, involved in homologous recombination and DNA repair, thereby providing an increased mutational burden that synergizes with PARP inhibition. Yet, in cancers in which BRCA1/2 is prevalent, PARP inhibitors have also shown an effect in the all-comer population. PARP inhibitors are increasing their importance in cancer therapy, and it is beyond the scope of the present manuscript to review them. It should be noticed, though, that although CRC is not yet an approved indication of any PARP inhibitor, several manuscripts have highlighted their potential, in particular in combination with DNA damaging agents such as irinotecan (Davidson et al., 2013; Genther Williams et al., 2015; Augustine et al., 2019), 5-fluorouracil and oxaliplatin (Jarrar et al., 2019), PI3K inhibitors in combination with anti-CTLA4 immunotherapy and X-ray radiation (Landry et al., 2020), p53 inhibitors (idasanutlin or pifithirin-β) (Smeby et al., 2020) or ATM inhibitors (Wang et al., 2017a). Mauri et al. have highlighted the “biomarkers of PARPness” which includes BRCA1/2 mutations in solid tumours, the sensitivity to platinum agents, but also ATM and CHK1 loss (Wang et al., 2017b; Mauri et al., 2020) (Table 1).

**TABLE 1:** PARP inhibitors in different cell models.

PARP inhibitor	Combinatory drug	Cell model	References
Veliparib (ABT-888)	Irinotecan (SN-38)	HCT-116	Davidson et al. (2013)
Olaparib	ATM inhibitor (KU55933)	SK-CO-1 and HCT 116	Wang et al. (2017b)
Rucaparib	Irinotecan	HCT-116	Augustine et al. (2019)
Talazoparib, niraparib	Idasanutlin (MDM2i) and pifithrin-β (p53i)	SK-CO-1, LS513, SW1222 and SNU61	Smeby et al. (2020)
Niraparib	HS-173 (PI3Ki) ± γH2AX or αCTLA-4	CT-26	Landry et al. (2020)
Niraparib	Irinotecan (SN-38)	Microsatellite stable/instable CRC cell lines	Genther Williams et al. (2015)
Olaparib	5-FU or oxalilplatin	CRC-patient-derived cell lines	Jarrar et al. (2019)

Different PARP inhibitors have already entered clinical trials in CRC (Table 2; clinicaltrials.gov). Several of them are still recruiting patients but others have already published encouraging results, despite the advanced settings. Pishvaian at al. have highlighted the efficient combination of veliparib and temozolomide in metastatic CRC population, refractory to other therapies, with a median OS of 6.6 months. More recently, Plummer et al. have demonstrated the efficacy of E7449 (PARP/tankyrase inhibitor) in patients with solid tumours, including CRC, showing a promising antitumoral activity with 50–800 mg oral dose. Furthermore, Chen et al. have evaluated that the combination of olaparib and irinotecan have appeared to be beneficial with intermittent administrations and dose reduction, avoiding side effects of both the molecules. On the other hand, Goburnova’s and Leichman’s groups have guided the only two terminated clinical trials in which, veliparib plus FOLFIRI and olaparib respectively, do not appear to be beneficial comparing to FOLFIRI alone or as a single agent. The conclusions of these studies are supported by the controlled nature of the trial, but both enrolled a very small number of patients in phase II trials. It is likely, therefore, that larger trials are needed to understand the potential of PARP inhibitors in CRC. For this to be successful, though, the correct patient population (in particular referring to the DNA repair machinery) and combination strategy should be optimized first.

**TABLE 2:** PARP inhibitors in clinical trials.

Study number	Phase	Drugs	Enrolment	Ref/Stage
NCT01051596	2	Veliparib + temozolomide	75 CRC patients incurable by surgery	Completed (Pishvaian et al., 2018)
NCT00912743	2	Olaparib	33 CRC patients incurable by surgery	Completed (Leichman et al., 2016)
NCT02484404	1/2	Cediranib+Olaparib+MEDI4736 (anti-PD-L1)	Phase 2 part of the study requests the participants to have tumor samples removed	Recruiting (clinicaltrial.gov)
NCT03875313	1/2	Talazoparib + telaglenastat (glutaminase inhibitor)	33 between different types of solid tumour	Terminated (clinicaltrial.gov)
NCT02305758	2	Velparib + FOLFIRI + bevacizumab	130 untreated metastatic colorectal cancer	Completed (Gorbunova et al., 2019)
NCT03337087	1/2	5-fluorouracil+leucovorin+liposomal irinotecan + rucaparib	CRC (up to 3 lines of prior therapy)	Recruiting (clinicaltrial.gov)
NCT03761914	2	Olaparib+durvalumab	Advanced Mismatch Repair Proficient Colorectal *Cancer* (MMRp-CRC)	Active (clinicaltrial.gov)
NCT04171700	2	Rucaparib	Mutations in Homologous Recombination Repair (HRR) genes	Recruiting (clinicaltrial.gov)
NCT01618136	1/2	E7449 (PARP/tankyrase inhibitor) + temozolomide or carboplatin and paclitaxel	41 patients with solid tumours	Completed (Plummer et al., 2020)
NCT00535353	1	Olaparib + irinotecan hydrochloride	26 patients with locally advanced or metastatic colorectal cancer	Completed (Chen et al., 2016)
NCT04456699	3	Olaparib+bevacizumab	Metastatic colorectal cancer (CRC) who have not progressed following first-line induction of FOLFOX with bevacizumab	Recruiting (clinicaltrial.gov)
NCT04166435	2	Olaparib + temozolomide	patients with MGMT promoter hypermethylated advanced colorectal cancer	Recruiting (clinicaltrial.gov)

### Therapeutic Role of Sirtuin Inhibitors in Colorectal Cancer

Another class of NAD-consuming enzymes that has a role in genomic stability, metabolism and senescence are SIRT1-7 (Vassilopoulos et al., 2011). Sirtuins are class III histone deacetylase which are highly conserved and use NAD as an acetyl acceptor leading to O-acetyl-ADPR and free nicotinamide. While first described as histone deacetylates, these enzymes have multiple targets and is therefore not surprising that the different isoforms shown different subcellular localization: SIRT1, SIRT6 and SIRT7 are mostly localised in the nucleus; SIRT3, SIRT4 and SIRT5 are in mitochondria; while SIRT2 is restricted to the cytoplasm (O’Callaghan and Vassilopoulos, 2017). They exert different activities, summarized in Table 3. All the different isoforms have been studied as putative prognostic biomarkers in CRC, and some of them, as SIRT1 and 2 have been considered papabile targets (Huang et al., 2016; Zu et al., 2016).

**TABLE 3:** SIRT properties in CRC.

Isoform	Localization	Properties in CRC	Inhibitor
SIRT1	Nucleus/Cytoplasm (O’Callaghan and Vassilopoulos, 2017)	Promotes EMT and metastasis formation (Cheng et al., 2016)	4bb, evodiamine, MHY2256b (Ghosh et al., 2017; Zhou et al., 2019; Kim et al., 2020)
SIRT2	Cytoplasm/Nucleus (O’Callaghan and Vassilopoulos, 2017)	Promotes tumour angiogenesis (Hu et al., 2018)	MHY2256, AK-1, AF8, AF10, AF12 (Cheon et al., 2015; Farooqi et al., 2019; Kim et al., 2020)
SIRT3	Mitochondria (O’Callaghan and Vassilopoulos, 2017)	Modulates mitochondria fission, mobility and proliferation (Wang et al., 2018a, 3)	-
SIRT4	Mitochondria (O’Callaghan and Vassilopoulos, 2017)	Increased sensitivity to -FU (Huang et al., 2016) and tumour-suppression (Miyo et al., 2015)	-
SIRT5	Mitochondria/Cytoplasm (O’Callaghan and Vassilopoulos, 2017)	-	-
SIRT6	Nucleus (O’Callaghan and Vassilopoulos, 2017)	Prognostic favourable (Zhang et al., 2019)	-
SIRT7	Nucleus (O’Callaghan and Vassilopoulos, 2017)	Radiosensitivity in CRC (Tang et al., 2017)	-

Several sirtuin inhibitors have been developed through the years, but none has so far entered clinical trials for cancer. The most studied inhibitors developed are against SIRT1 and SIRT2. Ghosh et al. designed a SIRT1 inhibitor named 4bb which is able to induce HCT116 apoptosis via p53-acetylation and Bax and caspase overexpression (Ghosh et al., 2017). Moreover, also MHY2256, a SIRT1/2 inhibitor has been shown to reduce viability of both p53 wild-type or mutant colorectal cancer lines (HCT116, HT29 and DLD-1) (Kim et al., 2020). Given that SIRT2 is thought to promote vascular endothelial growth factor A (VEGF-A) signalling and endothelial-like tube formation in tumour angiogenesis (Hu et al., 2018), specific inhibitors have also been developed. For example, AK-1, a cell-permeable benzylsulfonamide, is able to induce Snail down-regulation and the consequent block in G1-phase of HCT116 cell line (Cheon et al., 2015), while several lysine-based thioureas named AF8, AF10 and AF12 are able to counteract viability in HCT116 cell line and in the xenograft murine model (Farooqi et al., 2019).

No other inhibitors have so far been tested in CRC, but it is possible that also other sirtuins might be promising therapeutic targets.

### Therapeutic role of CD38 Inhibitors/Antibodies in Colorectal Cancer

CD38 is an ADP-ribosyl glycohydrolase mainly expressed in hematopoietic and non-hematopoietic cells, orchestrating activation and differentiation. CD38 is a transmembrane protein, localised on the cell membrane endowed of both enzymatic and receptor activity. As an ectoenzyme, it promotes the catabolism of extracellular NAD and NADP into ADPR or, in smaller amounts and depending on pH conditions, into the Ca2+-mediating second messengers cyclic adenosine diphosphate ribose (cADPR) and NAADP (van de Donk et al., 2018). The reaction produces cADPR, ADP ribose, and NAADP that stimulate Ca2+ mobilization (Malavasi et al., 2021). Whether in cancer biology this enzymatic activity is important has never been ascertained. On the other hand, as a receptor, CD38 is responsible of T cell activation, in particular in lamina propria cells, promoting colitis (Lischke et al., 2013) and it has been considered a positive prognostic marker for CRC (Perenkov et al., 2012).

Karakasheva et al. have highlighted that CD38 might be an interesting target for metastatic CRC. Using peripheral blood mononuclear cells (PBMCs) from CRC patients, compared to healthy donors, they observed an increased frequency of CD38^+^ monocytic-myeloid-derived suppressor cells (M-MDSCs) and CD38^+^ ppolymorphonuclear-myeloid-derived suppressor cells (PMN-MDSCs) compared to healthy monocytes, with the ability to induce immunosuppressive properties (Karakasheva et al., 2018). Moreover, CD38^+^M-MDSCs and CD38^+^PMN-MDSCs of patients who have previously undergo treatment are increased compared to naïve patients. Other Authors have highlighted the promising use of anti-CD38 antibodies in CRC patients refractory to other therapies, nonetheless no clinical trials or approvals have been proposed for CRC, even though anti-CD38 antibodies have represented a break-through in multiple myeloma (Nooka et al., 2019) and are at present approved in this indication.

### *Therapeutic Role of* Aldehyde Dehydrogenases *in Colorectal Cancer*

Among the different NAD-consuming enzyme, the aldehyde dehydrogenase (ALDHs) superfamily, composed by 19 different members, is known to be essential in the irreversible oxidation of a wide range of endo- and xenobiotic aldehydes to the respective carboxylic acid (Vassalli, 2019). Briefly, upon the activation of the catalytic cysteine (C302, numbering based on the ALDH2 sequence) by the glutamic acid (G268, numbering based on the ALDH2 sequence), the thiolate group interacts with the carbonyl carbon of the target aldehyde. After the deacylation, the hydride is transferred from the tetrahedral intermediate to the NAD pyridine ring. Finally, the thioester intermediate is hydrolysed, the reduced cofactor is dissociated and the enzyme can bind a new NAD molecule (Marchitti et al., 2008). The catalytic activity of ALDHs is essential to counterbalance the intrinsic toxicity of aldehydes, an extremely reactive chemical species extremely whose accumulation leads to DNA alkylation (LoPachin and Gavin, 2014). ALDHs are also involved in drug metabolism, and, for example, the active metabolite of cyclophosphamide, is a substrate of these enzymes (Tomita et al., 2016).

The relevance of this enzyme in the context of gastroenteric tumour is also given by the fact that CRCs are at times linked to high consumption of alcohol (Ferrari et al., 2012; Na and Lee, 2017). This could in part be attributable to acetaldehyde accumulation, produced by the alcohol oxidation (Zhang and Fu, 2021). Indeed, an unhealthy lifestyle with high alcohol consumption can be considered as one of the most common and important causes related to the potential development of CRCs and other significant cancers as head and neck, oesophageal and gastric (Zhang and Fu, 2021). Besides, a higher risk of CRCs has been demonstrated in Asian populations affected by a genetic polymorphism on the ALDH isoform 2, the key enzyme in acetaldehyde metabolism. The ALDH2*2 allele is extremely common in Est Asians, with the glutamine 487 substituted by a lysine (E487K) which intrinsic activity is reduced from 60 to 100% (Chang et al., 2017; Rossi et al., 2018; Wang et al., 2018b). Based on these suggestions, a small molecule activator of the enzyme, known as alda-1, has been developed and characterized. This molecule increases the catalytic activity of wild-type ALDH2 just two-fold but increases 11-fold the activity of homozygous ALDH2*2 (Chen et al., 2014). The high-resolution crystal structure of the ALDH2*2-Alda1 complex describes the peculiar mechanism of interaction in which the small ligands can reconstitute the correct folding at the level of the αG structure of the Rossman-fold essential for the interaction with the NAD^+^ adenosine ring and for the maintenance of the correct oligomerization, allowing the recovery of the full enzymatic activity (Perez-Miller et al., 2010).

Alongside activators in selected genetic contexts, inhibitors have also been proposed as possible therapeutics in CRC, due to an increased expression and activity of ALDHs (Khoury et al., 2012; Li et al., 2018; van der Waals et al., 2018; Yang et al., 2018; Attia et al., 2020). Interestingly, this is also true in the cancer stem cells (CSCs) (Clark and Palle, 2016), that are known as the most challenging to be targeted, refractory to common chemotherapeutics. It is also likely that this increased expression leads to chemotherapy and radiotherapy resistance of cancer cells and CSCs (Januchowski et al., 2013; Kim et al., 2017; Dinavahi et al., 2019; Muralikrishnan et al., 2020; Rebollido-Rios et al., 2020). ALDHs inhibitors have been proposed as monotherapies as well as in combination or as a tool for the early diagnosis (Koppaka et al., 2012; Anorma et al., 2018; Kim D. et al., 2018; Ferraris et al., 2020; Gelardi E. et al., 2021).

Among the 19 different isoforms, ALDH1A1 and 1A3, two key enzymes that participate in retinaldehyde oxidation (Moretti et al., 2016), have been scrutinized closely in several solid tumour, included the CRCs. Kovoska et al. have shown that abrogating *ALDH1A3* by siRNA-mediated gene silencing increases raltitrexed and 5-fluorouracil sensitivity of HCT-116 (Kozovska et al., 2018). Similarly, our group has recently described new ALDH1A3 inhibitors with a conserved imidazo [1,2- α]pyridine ring scaffold, considered as synthetic analogues of daidzin, a known reversible inhibitor of the ALDH isoenzymes (Quattrini et al., 2020b; Quattrini et al., 2020a). In particular one of the most promising compounds, called NR6, showed a strong potency and selectivity both against recombinant enzymes and cells overexpressing ALDH1A3 (Gelardi E. L. M. et al., 2021). The *in vitro* treatment with NR6 led to a strong cytotoxic effect only in cancer cell lines (HCT-116) overexpressing the target protein without inducing any significant toxicity in non-cancerous fibroblasts (CCD-18Co). The role of ALDH1A1 in CRCs is still unclear, outside the capacity to reduce the oxidative stress and the activity of alkylating agent, but it is considered as promising marker for the characterization of tumour malignancy and to locate the cancer stem cells (Ciccone et al., 2020). A small series of ALDH1A1 and ALDH1A pan-inhibitors are available, called CM10, A37 and NCT 501 hydrochloride (Morgan and Hurley, 2015; Yang et al., 2015; Chefetz et al., 2019) that were deeply investigated against ovarian cancer (Nwani et al., 2019), but that could be also useful to characterize the potential validity of ALDH1A1 as target in CRCs.

Another isoform that has gained interest in the past few years is mitochondrial ALDH1B1, the second most important enzyme in the alcohol metabolism, highly expressed in the crypts of intestinal tissues and a possible actor for tumour progression (Langan et al., 2012; Singh et al., 2015). Also in this case, immunohistochemistry experiments have shown a higher expression levels in cancer stem cells (Chen et al., 2011).

Taken together, these data suggest the relevance of this enzymatic superfamily as an intriguing and innovative target for cancer therapy, not only from the common perspective of the small inhibitor development but also as a marker for the early diagnosis.

## NADPH Synthesis as a Target

**FIGURE 2:**
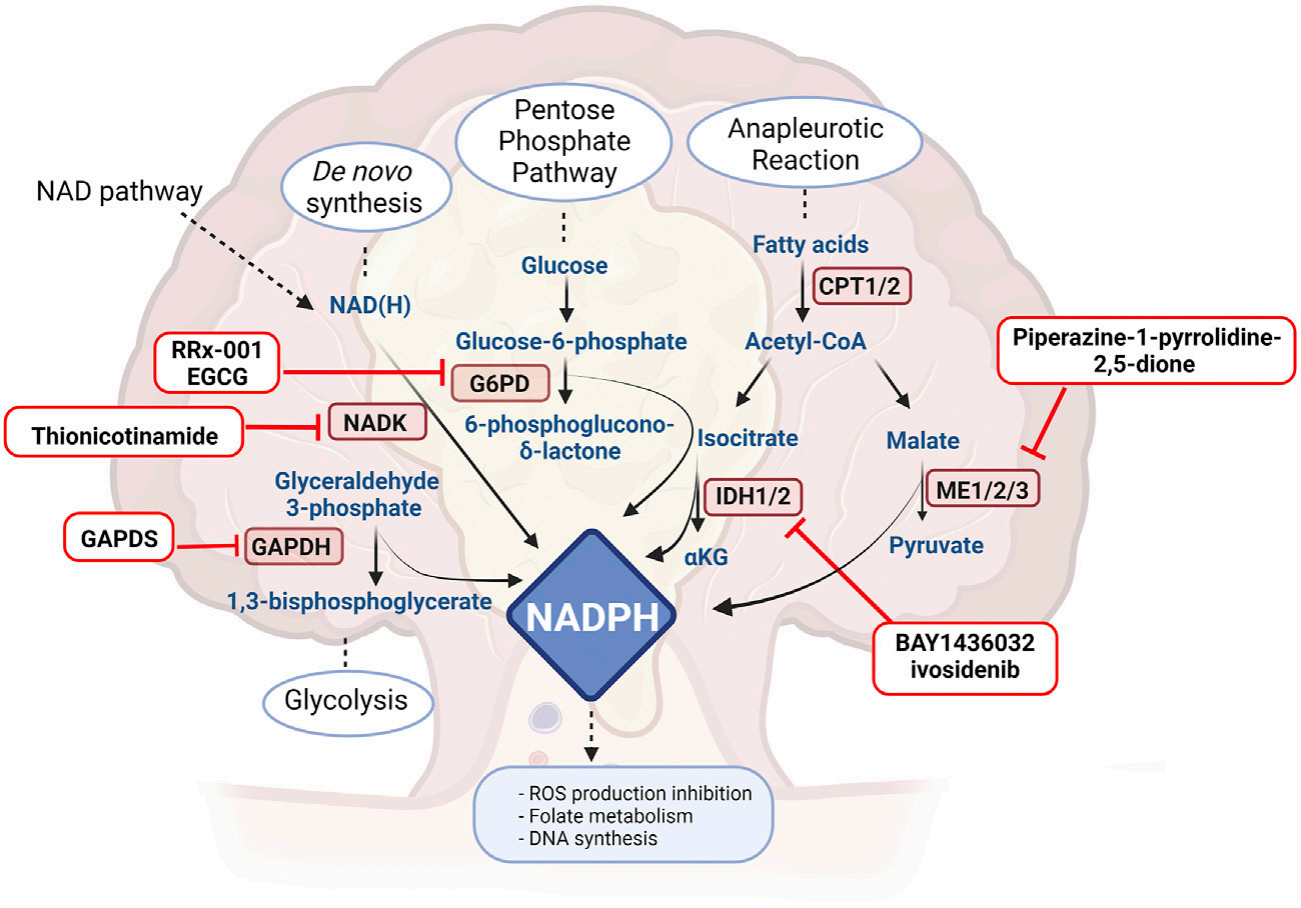
Drugs or molecules that have been postulated as effective in CRC and that target NADPH routes. G6PH: glucose 6-phosphate dehydrogenase, NADK: NAD kinase, CPT: carnitine palmitoyl transferase, ME: malic enzyme, IDH: isocitrate dehydrogenase, GAPDH: glyceraldehyde 3-phosphate dehydrogenase. Created with BioRender.com.

As mentioned above, ROS production may determine DNA damage and apoptosis. *Cancer* cells have the ability to scavenge ROS formation by activating antioxidant mechanisms, but usually these require high NADPH levels (Ju et al., 2020). There are several mechanisms orchestrated by NADPH balance, including antioxidant reactions that lead to reduced glutathione, necessary for hydrogen peroxide reduction and fatty acids, amino acids and nucleotides synthesis able to promote tumour growth (Ju et al., 2020; Rather et al., 2021). Moreover, NADPH works as an essential electron donor and cofactor maintaining reduction potential in anabolic reactions. In cancer cells, NADPH levels are controlled by several pathways, with the aim not only to contrast ROS production, but also in order to promote several metabolic reactions able to induce tumour proliferation, including NAD kinase (NADK), malic enzymes (ME) and NADP-dependent isocitrate dehydrogenases (IDH1 and IDH2) (Ju et al., 2020) but also glyceraldehyde 3-phosphate dehydrogenase (GAPDH). Also in this scenario, a number of agents have been developed and proposed to contrast NADPH homeostasis in cancer (Figure 2).

### Therapeutic Role of NADK Inhibitors in Colorectal Cancer

*De novo* synthesis of NADPH is orchestrated by NADK, localised in mitochondria and in the cytosol, which phosphorylates NAD to NADP (Katsyuba et al., 2020; Pramono et al., 2020). Cytosolic NADK is overexpressed in several tumours, including colorectal cancer (Ju et al., 2020; Pramono et al., 2020). More importantly, cancer cells bear NADK mutations endowed of increase enzymatic activity, inducing higher production of NADPH and reduction of ROS, sustaining cancer cell viability (Tedeschi et al., 2016). It is well known that the silencing of NADK impairs cancer growth in human colorectal cancer (Tedeschi et al., 2015; Yau et al., 2017), but only one inhibitor has so far been described. Thionicotinamide, a structural analogue of NAD, is able to counteract the growth of C85 (human colorectal cell line) both *in vitro* and *in vivo*, impairing the NADPH pool and augmenting ROS production (Tedeschi et al., 2015). The possibility to target only the mutated form of NADK is intriguing, but at present no specific inhibitor has been described.

### Therapeutic Role of Malic Enzymes in Colorectal Cancer

Malic Enzymes contribute to both anabolic and catabolic reactions. They are tetrameric proteins with a double dimer structure, acting as oxidative decarboxylases. Malic enzymes are responsible for different actions which includes the decarboxylation of malate into pyruvate using NADP and producing NADPH, into Krebs cycle. Malic enzymes display different localisations: malic enzyme 1 (ME1) is in cytosol while malic enzyme 2 (ME2) and malic enzyme 3 (ME3) are located in mitochondria. ME1 participates in glycolysis and Krebs cycle but also in fatty acid and cholesterol synthesis using NADPH. ME1 produces NADPH stocked in the cytosol, which will be used by fatty acid synthase. (Fernandes et al., 2018).

Increased expression of MEs or increased activity has been associated to several types of cancer (Loeber et al., 1994; Ju et al., 2020) and all three isoforms have been associated with a worse prognosis in several type of cancer. In CRC, the literature has focused its attention on ME1, that, as other NADPH-dependent enzymes, also maintains the redox homeostasis in cells (Ju et al., 2020). Fernandez et al. have shown the importance of ME1 in the development of the tumoral mass in APCmin/+ mice. In detail, the number and the area of adenomas are increased in mice overexpressing ME1, which directly activates and sustains the Wnt/β-catenin pathway. Moreover, silencing ME1 in several CRC cell lines determines cell death and senescence (Murai et al., 2017). Furthermore, using piperazine-1-pyrrolidine-2,5-dione, a non-specific inhibitor (Zhang et al., 2006), they observed a decreased viability of HCT116 and HT29 treated or not in combination with a Wnt-canonical pathway inhibitor, confirming the potential of MEs as drug targets (Fernandes et al., 2018; Ju et al., 2020).

### Therapeutic Role of G6PD in Colorectal Cancer

The pentose phosphate pathway (PPP) is among the principal cellular NADPH suppliers. The PPP is divided into an oxidative branch in which glucose-6-phosphate dehydrogenase (G6PD) converts glucose-6-phosphate into pentose phosphate metabolites, synthetizing NADPH, and in a non-oxidative branch that recycles pentose phosphates to glycolytic intermediates or produces ribose-5-phosphate (Boren et al., 2002). G6PD has been found over-expressed and over-activated in several cancers, determining poor prognosis and chemoresistance (Zhang et al., 2017; Yu et al., 2019; Ju et al., 2020), while its deficiency appears to decrease predisposition to cancer (Pes et al., 2019).

Several G6PD inhibitors have been developed. In CRC, RRx-001 has been broadly studied (Reid et al., 2014; Oronsky et al., 2016). A comparative clinical trial in phase 2 (NCT02096354) is active comparing RRx-001 in metastatic colorectal cancer in combination with irinotecan. Moreover, epicatechin gallate (EGCG), a putative G6PD inhibitor, has also been investigated in CRC, and has been shown to reduce the *de novo* synthesis of fatty acids and the pentose phosphate pathway in HT29 cells (Sánchez-Tena et al., 2013). EGCG has entered an early phase 1 clinical trial (NCT02891538) as chemo-preventive drug in patients who have undergone surgery and who do not require treatment after resection because of a benign prognosis.

### Therapeutic Role of Isocitrate Dehydrogenase in Colorectal Cancer

Isocitrate dehydrogenase, an enzyme involved in TCA, synthetizes isocitrate from α-ketoglutarate, converting NADP to NADPH (Clark et al., 2016). It is present in three different isoforms: IDH1, situated in the cytosol and in peroxisomes, IDH2 and IDH3, which are located in mitochondria (Pramono et al., 2020). Both IDH1 and IDH2 are overexpressed in several types of tumours, decreasing ROS production and increasing concomitantly NADPH. Targeting IDH1 seems to be appealing in both wild-type and mutant forms. Indeed, IDH1 and IDH2 mutations occur in several tumours (Whitehall et al., 2014). In particular, the R132H IDH1 mutation lacks the classical enzymatic activity and is endowed of a particular synthetic pathway in which α-ketoglutarate is converted to 2-hydroxyglutarate. 2-hydroxyglutarate appears to induce cell proliferation via the mTOR-signalling pathway (Neitzel et al., 2020) and increases lipogenesis during hypoxia (Reitman et al., 2014). The mutated IDH1^R132H^ substantially decreases NADPH production (Bleeker et al., 2010). Tougeron et al. have not manged to observe IDH1/2 in CRC patients (Tougeron et al., 2016), while Whitehall et al. have underlined that these mutation are present in correspondence to the CpG island methylator phenotype and in the presence of BRAF mutations (Whitehall et al., 2014). The idea of targeting both the wild-type form or the mutated form appears to be beneficial in CRC (Neitzel et al., 2020). IDH1 mutations appear to be important also in IBD-CRCs where sporadic cancer gene mutations occur less often. In this case, targeted IDH therapy appears to be helpful (Alpert et al., 2019). Moreover, 2-hydroxyglutarate has been found elevated also in presence of wild-type IDH, in HCT116 and RKO cell lines, responsible of epithelial-mesenchymal transition and development of metastases (Fallah-Rad, 2016). On the other hand, wild-type IDH1 silencing determines NADPH reduction and the consequent sensibilization to chemotherapy (Ju et al., 2020), while HCT116 cell line, endowed of the mutated form, are more sensitive to cisplatin treatment, and this is reverted by AGI-5198, an inhibitor of IDH1 mutated form (Khurshed et al., 2018). In contrast, the use of the IDH1 inhibitor BAY1436032 in mutant HCT116 (on R132H) leads to cell death (Pusch et al., 2017). Another important evidence is that the inhibitors of IDH mutated enzymes, as ML309 in combination with vitamin C, are responsible for the decrease of 2-hydroxyglutarate. This reduction determines an increase of DNA hydroxymethylation (Gerecke et al., 2020). What is also important is the post-translational modifications on IDH1. Wang et al. have highlighted that IDH1 is hyperacetylated at lysine 224, promoting tumoral expansion and metastases, sign of poor prognosis. SIRT2 is responsible of the deacetylation of lys224 in IDH1, increasing IDH1 enzymatic activity and reducing liver metastases (Wang et al., 2020). In conclusion, targeting mutated IDH in CRC seems to be appealing for IBD-CRCs, while in sporadic CRC wild-type IDH seems to be more plausible. Several mutant IDH1 inhibitors have entered clinical trials for hematologic malignancies (Rather et al., 2021), while only ivosidenib has been proposed as wild-type inhibitor in myeloid neoplasms (NCT03564821). The only clinical trial proposing IDH1 inhibitor for CRC is NCT04584008, aiming to use DNA sequencing in order to enrich patient populations with selected genotypes.

### Therapeutic Role of GADPH in Colorectal Cancer

Glyceraldehyde 3-phosphate dehydrogenase is an enzyme that catalyses the sixth step of glycolysis and, surprisingly, is also a moonlighting protein, *i.e*., a protein that possesses other cellular functions alongside its prototypical one. Given the relevance in glycolytic metabolism it has obviously been linked to the energy metabolism of cancer cells. A genome-wide microarray analysis has revealed that GAPDH is highly expressed in rapidly proliferating colon cancer cells. Moreover, the glycolytic inhibition with Na iodoacetate promotes *in vitro* the reduction of growth of different colorectal cancer cells (Bazzocco et al., 2015) and the regression of xenograft tumours inducing necrosis (Sánchez-Aragó and Cuezva, 2011).

Given that GAPDH nitrosylation has been demonstrated to trigger nuclear translocation and initiate p53-mediated cell death (Hara et al., 2005), a recent article has provided the mechanism by which microcystins-LR (MC-LR), a toxin produced by cyanobacteria, induces colon cancer cells apoptosis. MC-LR cytotoxicity is associated with nitric oxide (NO) increases that induce GAPDH nitrosylation with the consequent nuclear translocation and colorectal cancer cell p53-mediated apoptosis (Li et al., 2020). Other evidence suggests that nuclear translocation of GAPDH increases under stressful stimuli. Grolla and collaborators have indeed demonstrated that GAPDH can be the shuttle for NAMPT from the cytosol in the nucleus under stress condition as oxidative stress, NO-induced stress and DNA damage to sustain the NMN/NAD pool (Grolla et al., 2016). Yet, the inhibitor of GAPDH shuttling to the nucleus, omigapil (Erb et al., 2009), has been evaluated in neurodegenerative disorders but has had little success in the cancer field.

Among several catalytic inhibitors, the triazine-based small molecule GAPDS has been reported to exert a specific anticancer activity in human carcinoma cells by preventing GAPDH tetramerization. GAPDS reduces cell viability in both normoxic and hypoxic conditions with an enhanced expression of apoptotic markers. Moreover, the effects on tetramerization result in a reduction in cytosolic GAPDH and tubulin expression which have a direct impact on the ability of cancer cells to invade and migrate (Jung et al., 2014). The involvement of GAPDH on cell motility has also been confirmed (Liu et al., 2017).

Studies on patient samples confirm the involvement of GAPDH in colorectal cancer. GAPDH mRNA levels were shown to have a 1.6-fold expression increase between normal and colorectal cancerous tissues and 2.3-fold expression comparing colorectal liver metastases and liver tissues (Rubie et al., 2005). A study on vitamin C on human colorectal cancer cells suggests that ROS are implicated in the mechanism by which vitamin C inhibits GAPDH. Vitamin C intracellular accumulation leads to the production of endogenous ROS, resulting in the reduction of GAPDH activity via post-translational modifications and NAD depletion (Yun et al., 2015).

Despite one study shows a reduction of GAPDH expression in presence of metastasis (Tarrado-Castellarnau, 2017) some contrasting evidence also suggests a relationship between motility of cancer cells and this protein. Indeed, an increased expression of GAPDH in human colorectal cancer cells was associated with epithelial to mesenchymal transition (EMT) accompanied by the upregulation of mesenchymal markers (Liu et al., 2017). EMT is an important process involved in tumour invasion and metastasis formation and requires several regulators. The zinc-finger protein Snail binds to E-cadherin, suppresses the expression of the adhesion molecule and promotes the phenotypic transition (Cano et al., 2000). The silencing of GAPDH was shown to revert this phenomenon downregulating Snail expression associated with a reduction of vimentin and an increased expression of E-cadherin. Chromatin immunoprecipitation reveals that GAPDH physically interacts with the transcriptional factor Sp1 that binds to Snail promoting EMT (Liu et al., 2017). A recent study demonstrates the toxicity of a lipopeptidyl benzophene, asperphenin B, on human colorectal cancer cells associated with the downregulation of GAPDH expression. Moreover, GAPDH upregulation was found in metastasized cells and the anti-metastatic activity of the compound *in vitro* was correlated with the modulation of EMT signalling pathways (Byun et al., 2021).

GAPDH expression levels, considering all the stages of tumour progression, were not correlated with survival in patients with colorectal cancer (Tarrado-Castellarau, 2017) despite the evidence are not univocal.

Barbazán et al., have reported a multimarker expression panel of circulating tumour cells (CTC) to forecast prognosis in patients with metastatic colorectal cancer both at baseline and during treatment to monitor therapy response. GAPDH expression levels were included in the six-gene panel markers and Kaplan-Meier plots reveal an inverse correlation between high CTC group, progression-free survival and overall survival, elucidating an increased risk of tumour progression (Barbazán et al., 2014).

Therefore, GAPDH expression in colorectal cancer can contribute to highlight mechanisms involved in tumour progression and univocal data on patients will prompt the development of GAPDH targeted therapy in the clinical set.

## Conclusion

The aim of this review was to understand how NAD and NADPH routes may be important in CRC progression, highlighting the enzymes that might represent pharmacological targets. NAD biosynthesis, the use of NAD as an ADPR donor and NADPH homeostasis are crucial for cellular metabolism and signalling. Not surprisingly, therefore, these pathways are upregulated in cancer, and this is true also for CRC. Yet, few of the players of the NADome have been investigated thoroughly in this setting, despite an important unmet therapeutic need. NAMPT and PARP at present appear to be the most promising targets. iNAMPT inhibitors have possibly been abandoned too early, for on target side effects, without giving enough emphasis on the identification of the patient population that might benefit more from these treatments, thereby generating a favourable benefit/risk. This may possibly be given by understanding how to determine the NAMPT-dependence of tumours. Moreover, the evidence on eNAMPT deriving from different settings support a potential beneficial effect of eNAMPT neutralization also in CRC.

PARP inhibitors have entered several clinical trials, primarily in combination. For some of the concluded trials, the combination of a PARP inhibitor with other molecules (e.g., irinotecan) appears to be therapeutically superior compared to classical alkylating agents, while the outcome of the other trials is awaited.

Last, the other NAD and NADPH routes targets reviewed by us appear to be either CRC markers or promoters of CRC progression. Yet, specific molecules to unravel their potential in CRC treatment have yet to be disclosed. SIRT, G6PD and ALDH inhibitors have never entered clinical trial, but their ability to contrast CRC development, as highlighted by our study, prompts the investigation of the molecules that will arise in this setting. Importantly, the innovative IDH inhibitor ivosidenib, that has a wild-type inhibitor active in myeloid neoplasms, may also be an alternative to be tested in IBD-CRCs, where IDH is usually not mutated.
